# ERRα activates SHMT2 transcription to enhance the resistance of breast cancer to lapatinib via modulating the mitochondrial metabolic adaption

**DOI:** 10.1042/BSR20192465

**Published:** 2020-01-17

**Authors:** Xin Li, Kejing Zhang, Yu Hu, Na Luo

**Affiliations:** Department of Breast Surgery, Xiangya Hospital, Central South University, Changsha, Hunan 410008, China

**Keywords:** breast cancer, ERRα, lapatinib, mitochondrial metabolic adaption, resistance, SHMT2

## Abstract

Lapatinib, a tyrosine kinase inhibitor, can initially benefit the patients with breast tumors but fails in later treatment due to the inevitable development of drug resistance. Estrogen-related receptor α (ERRα) modulates the metabolic adaptations in lapatinib-resistant cancer cells; however, the underlying mechanism remains unclear. ERRα was predicted to bind to the serine hydroxymethyltransferase 2 (SHMT2) transcription initiation site in the ER- and HER2-positive cell line BT-474; thus, we hypothesize that ERRα might modulate the resistance of breast cancer to lapatinib via regulating SHMT2. In the present study, we revealed that 2.5 and 5 µM lapatinib treatment could significantly decrease the expression and protein levels of ERRα and SHMT2; ERRα and SHMT2 expression and protein levels were significantly up-regulated in breast cancer cells, in particularly in breast cancer cells with resistance to lapatinib. ERRα knockdown restored the inhibitory effects of lapatinib on the BT-474R cell viability and migration; in the meantime, ERRα knockdown rescued the production of reactive oxygen species (ROS) whereas decreased the ratio of glutathione (GSH)/oxidized glutathione (GSSG) upon lapatinib treatment. Via targeting SHMT2 promoter region, ERRα activated the transcription of SHMT2. The effects of ERRα knockdown on BT-474R cells under lapatinib treatment could be significantly reversed by SHMT2 overexpression. In conclusion, ERRα knockdown suppresses the detoxification and the mitochondrial metabolic adaption in breast cancer resistant to lapatinib; ERRα activates SHMT2 transcription via targeting its promoter region, therefore enhancing breast cancer resistance to lapatinib.

## Introduction

Breast cancer is one of the most common malignant tumors occurring on women throughout the world [1–3]. Breast cancers are characterized by genetic heterogeneity and classified according to their molecular properties. There are five subtypes of intrinsic breast cancer: luminal A, luminal B, normal breast-like, human epidermal growth factor receptor 2 (HER2)-enriched and basal-like, each of which is unique in terms of incidence, survival and therapeutic response [4].

The receptor tyrosine kinase (RTK) signaling pathway overexpression and abnormal activation are essential in the development of human breast carcinoma [5,6]. As a dual epidermal growth factor receptor (EGFR)/human EGFR-2 (HER2) tyrosine kinase inhibitor (TKI), lapatinib is approved for use in patients with metastatic HER2-amplified breast tumors [7,8]. Although lapatinib initially can benefit the patients with breast tumors, the inevitable development of drug resistance happens [9].

The metabolism of tumor cells influences their drug reaction. In fact, breast cancer cells with the resistance to lapatinib demonstrate an increase of genes that regulate the glucose-deprivation response network, indicating that cellular metabolism may influence the response to lapatinib [10]. Oestrogen-related receptor α (ERRα, NR3B1) is an orphan member of nuclear receptor superfamily [11] and a major regulatory factor of normal and tumor cell energy metabolism [12–14]. The expression of ERRα is positively correlated with HER2 status and bad prognosis within breast tumors [15,16]. More importantly, ERRα re-expression within drug-resistant cells activates metabolic adaptations favoring mitochondrial energy metabolism via up-regulated glutamine metabolism, and ROS detoxification that is necessary for cell viability under the treatment stresses. Inhibition of ERRα may serve as a potent auxiliary method for poorly treated HER2-positive breast carcinoma [9]. Developing an in-depth understanding of the mechanism by which ERRα modulates the metabolic adaptations in lapatinib-resistant cancer cells might extend its clinical applications.

Being a transcriptional mediator, ERRα could translate downstream mitogenic signals into metabolic signatures, which could be impaired upon the treatment of lapatinib, a RTK inhibitor [17]. Thus, we attempted to analyze mitochondrial metabolism-related genes that might be transcriptionally regulated by ERRα. The Spearman’s correlation analysis revealed that ERRα could be positively related to SHMT2 (*R* = 0.34, *P* < 0.001) based 526 cases of breast cancer patients in TCGA database. SHMT2 (serine hydroxymethyltransferase 2) is a mitochondrial gene involved in serine catabolism necessary for the normal mitochondrial translation initiation and the maintenance of formylmethionyl tRNA [18,19]. The clinical data analyses also identify SHMT2 expression as a dangerous factor for patients with breast carcinoma, of which the expression level is positively correlated with breast cancer grade [20,21]. High SHMT2 expression is considerably related to lower overall survival in patients with breast carcinoma [22]. More importantly, co-immunoprecipitation data (ChIP-Atlas/Enrichment Analysis) demonstrated that ESRRA binds to the SHMT2 transcription initiation site in the ER- and HER2-positive cell line BT-474. Based on these analyses, we hypothesize that ERRα might modulate the resistance of breast cancer to lapatinib via regulating SHMT2.

Herein, ERRα and SHMT2 expression and protein levels could be determined in parental BT-474 cells upon lapatinib treatment. Lapatinib-resistant BT-474R cell line was established and examined for the expression and protein levels of ERRα and SHMT2. The predicted binding between ERRα and SHMT2 was validated. The detailed effects of ERRα on SHMT2 expression, on the cell viability, migration capacity, the production of ROS, and the ratio of GSH/GSSG within breast cancer cells with or without resistance to lapatinib could be examined. Finally, we detected the dynamic effects of ERRα and SHMT2 to estimate whether ERRα modulates breast cancer cell resistance to lapatinib through SHMT2. In summary, the purpose of our study was to explore a novel regulatory mechanism of ERRα serving as a transcription factor to activate the transcription of SHMT2 and to affect ER- and HER2-positive breast cancer cell resistance to lapatinib.

## Materials and methods

### Cell line and cell transfection

BT-474 (ATCC® HTB-20™) cell line (HER2-positive and ER-positive) was obtained from the ATCC (Manassas, VA, U.S.A.) and cultured in RPMI1640 (Gibco, Waltham, MA, U.S.A.) medium supplemented with 10% fetal bovine serum (FBS, Gibco), penicillin (100 IU/ml) and streptomycin (100 mg/ml) at 37°C with 5% CO_2_.

Lapatinib-resistant BT-474R cells were developed from BT-474 cells by treatment with gradually increasing concentrations of lapatinib in cell culture medium for 6 months [23]. Cell viability assay showed that BT-474R cells could tolerate much higher concentrations of lapatinib compared with BT-474 cells, with their IC_50_ concentrations found to be about 4-fold higher than those of parental BT-474 cells [23,24].

ERRα silence or SHMT2 overexpression was conducted by the transfection of si-ERRα or SHMT2-overexpressing vector (GenePharma, Shanghai, China) with the help of Lipofectamine® 2000 Transfection Reagent (Thermo Fisher Scientific, Waltham, MA, U.S.A.).

For lapatinib treatments, with or without transfected cells were exposed to lapatinib (0.125, 0.25, 0.5, 1, 2, 2.5, 4, 5, 8, 16, 32 μM) for 24 h, cells were used for further experiments.

### Real-time PCR-based analyses

Total RNA was extracted using TRlzol® reagent (Life Technologies, Grand Island, NY, U.S.A.) according to the manufacturer’s instructions. The expression levels of target genes under different treatment conditions were assessed using SYBR green-based quantitative reverse transcriptase real-time polymerase chain reaction (qRT-PCR) (Yekta Tajhiz Azma, Tehran, Iran) taking GAPDH expression as an endogenous normalization.

### Immunoblotting

After transfection or lapatinib treatment, cells were lysed in RIPA buffer (Beyotime, shanghai, China). The protein levels were determined using immunoblotting analyses following a method described previously [25] with the following primary antibodies, anti-ERRα (ab76228, Abcam, Cambridge, MA, U.S.A.) and anti-SHMT2 (ab224428, Abcam), and a secondary anti-mouse antibody conjugated with horseradish peroxidase (Jacksons Immunoresearch, Mill Valley, CA, U.S.A.). GAPDH protein levels were detected with anti-GAPDH antibody (ab8245, Abcam) and used as an internal control to normalize the levels of target proteins.

### Cell viability determined by MTT assays

The cell viability and the inhibitory concentration 50% (IC_50_) of lapatinib was determined using an MTT assay following a method described previously [26]. Briefly, after transfection or lapatinib treatment, cells were incubated in the 100 μl fresh medium supplemented with 20 μl MTT solution (5 mg/ml) for 4 h at 37°C. At the end of incubation, the medium was gentle removed and 100 μl DMSO was added into each well. The optical density (OD) of each concentration was measured at 490 nm and IC_50_ was calculated using Prism v. 6.0 software (Graphpad Software, La Jolla, CA, U.S.A.). Cell viability inhibition rate by 24 h treatment of 5 μM lapatinib was calculated by [1-(OD 0h-OD 24h)/OD 0h] ×100%.

### Chromatin immunoprecipitation (ChIP)

To validate the predicted binding of ERRα to SHMT2 promoter region, the ChIP assay was performed following a method described previously [27]. A positive control antibody (RNA polymerase II), a negative control non-immune IgG and anti-ERRα were used. The immunoprecipitated DNA was subsequently cleaned, released, eluted and used for ChIP-PCR. The fold-enrichment (FE) was calculated as the ratio of the amplification efficiency of the ChIP sample to that of the non-immune IgG [27].

### Migration capacity determined by Transwell assay

The migration capacity of cells was determined using a Transwell assay following a method described previously [27]. After discarding the non-invasive cells, we fixed the invasive cells on the lower membrane surface, stained them with Crystal Violet solution (Beyotime Institute of Biotechnology, Haimen, China), and counted the cell number under a microscope.

### ROS production determined by flow cytometry

Target cells were cultured in 12-well plates at a density of 3 × 10^4^ cells/well for 12 h and then transfected and treated with 5 μM lapatinib for 24 h. Cells were incubated with 10 μM DCFH-DA for another 4h at at 37°C with 5% CO_2_. The cells were then harvested by trypsinization. The intracellular ROS was then determined using flow cytometry following the methods described before [28].

### GSH and GSSG determination

After treated with 5 μM lapatinib for 24 h, cells were collected after washed thrice with phosphate buffer. GSH and GSSG Assay Kit were purchased from Beyotime (Shanghai, China). The determination of GSH and GSSG was performed according to the manufacturer’s instructions.

### Mitochondrial membrane potential measurement

Mitochondrial membrane potential was assessed using 5, 50, 6, 60-tetrachloro-1, 10, 3, 30 tetraethylbenzimidazolecarbocyanide iodine (JC-1, Thermo Fisher Scientific). Briefly, the treated BT-474 and BT-474R cells were harvested by trypsinization and incubated with JC-1 staining solution (5 mg/ml) for 20 min at 37°C in the dark and rinsed twice with buffer. JC-1 fluorescence was determined by flowcytometry.

### Statistical analysis

All experimental data were presented as mean ± standard deviation. All *in vitro* experiments were independently performed at least three times. Comparison of data from two groups was performed via Student’s *t*-test when the data contended a normal distribution, otherwise Mann–Whitney *U*-test was adapted. One-way analysis of variance (ANOVA) followed by Tukey’s multiple comparison test was performed to compare these data between three groups or more groups. A *P* value less than 0.05 was considered to be statistically significant in the present study. GraphPad Software was used to analyze the data in the present study.

## Results

### ERRα and SHMT2 expression could be up-regulated by lapatinib and is higher in lapatinib-resistant breast cancer cells

**Figure 1:**
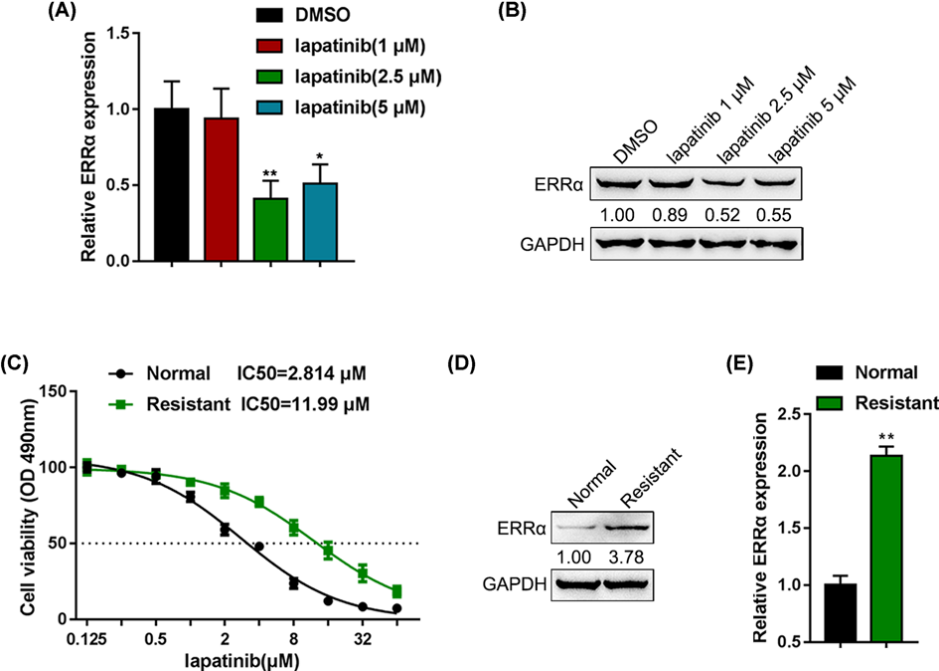
ERRα expression could be up-regulated by lapatinib and is higher in lapatinib-resistant breast cancer cells BT-474 cells were treated with lapatinib (0, 1, 2.5 and 5 µM) and examined for (**A**) the expression of ERRα by real-time PCR and (**B**) the protein levels of ERRα by Immunoblotting. (**C**) BT-474R cell line was established by treating BT-474 cells with gradually increasing lapatinib for 6 months. Then, MTT assay was performed to evaluate cell viability of BT-474 and BT-474R upon treatment with various concentrations of lapatinib (0.125, 0.5, 2, 8 and 32 µM) for 24 h. (**D**) The protein levels of ERRα determined in BT-474 and BT-474R cell lines by Immunoblotting. (**E**) The expression of ERRα determined in BT-474 and BT-474R cell lines by real-time PCR; *P < 0.05, **P < 0.01.

First, we treated BT-474 cells with lapatinib (0, 1, 2.5 and 5 µM) and examined the expression of ERRα. Figure 1A showed that the expression of ERRα could be significantly down-regulated by 2.5 and 5 µM lapatinib treatment; consistently, the protein levels of ERRα are decreased by 2.5 and 5 µM lapatinib treatment (Figure 1B).

To investigate the detailed functions of ERRα in breast cancer resistance to lapatinib, we first established lapatinib-resistant breast cancer cell line from parental BT-474R cells by treating the cells with gradually increasing concentrations of lapatinib for 6 months. We conducted the assay on cell viability to determine the resistance to lapatinib, and found that the IC_50_ of BT-474R cell line is significantly higher than that of BT-474 cell line (Figure 1C). Next, the protein levels and expression of ERRα were determined in BT-474 and BT-474R cells. Figure 1D,E showed that ERRα expression and protein levels were remarkably enhanced within BT-474R cell lines than those within parental BT-474 cell lines, indicating that ERRα might be a key factor in breast cancer cell resistance to lapatinib.

### ERRα modulates the migration capability, cell viability and mitochondrial metabolism in BT-474 and BT-474R cells

**Figure 2:**
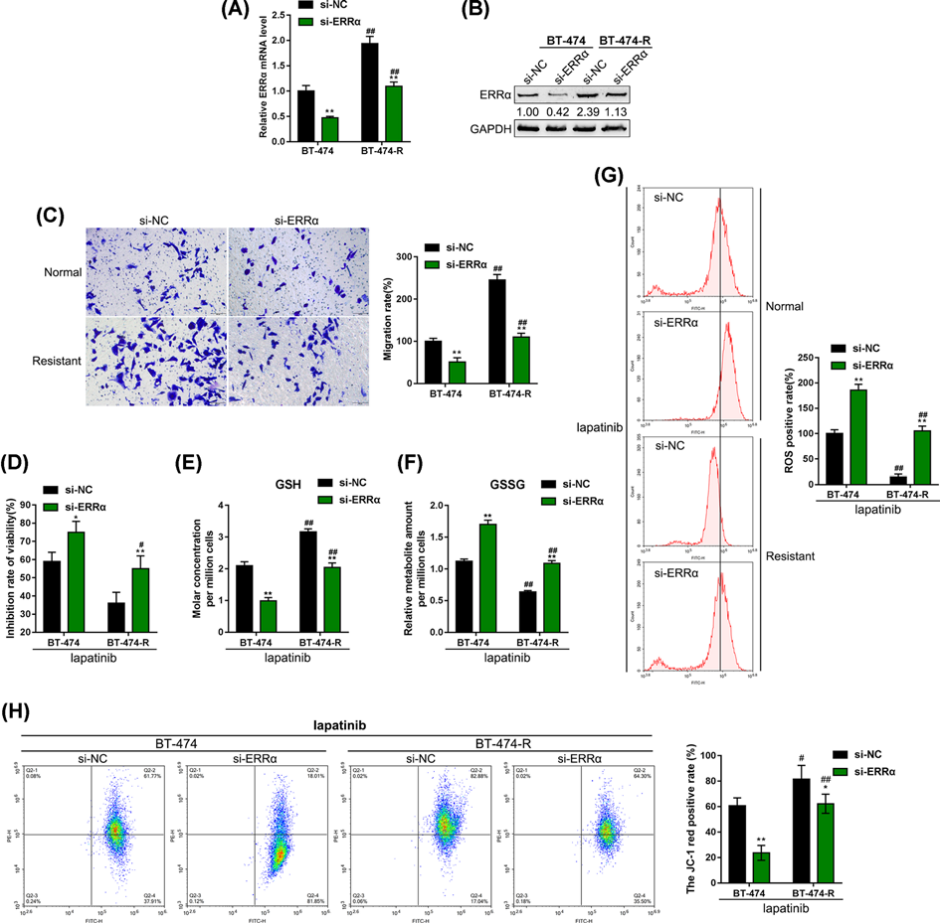
ERRα affects migration capability, cell viability and mitochondrial metabolism in BT-474 and BT-474R cells (**A**) ERRα knockdown was conducted in BT-474 and BT-474R cells by transfection of si-ERRα, as confirmed by real-time PCR. The expression of SHMT2 in response to ERRα knockdown was determined by real-time PCR. Then, BT-474 and BT-474R cells were transfected with si-ERRα and examined for (**B**) the protein levels of ERRα and SHMT2 by Immunoblotting. (**C**) The migration capacity by Transwell assay. (**D** and **G**) Under 5 μM lapatinib treatments, the inhibitory rates of cell viability were determined by MTT assays (**D**). The GSH and GSSG levels were determined by corresponding kits (**E** and **F**). The ROS production was measured by flow cytometry (G). (**H**) The mitochondrial membrane potential was detected by JC-1 staining; *P < 0.05, **P < 0.01, compared with si-NC group; #*P* < 0.05, ##*P* < 0.01, compared with parental BT-474 cells group.

Next, we investigated the detailed molecular effects of ERRα on breast cancer cells. We transfected si-ERRα to conduct ERRα knockdown within BT-474 and BT-474R cell lines, and performed real-time PCR and immunoblotting to verify the transfection efficiency (Figure 2A,B). After transfecting BT-474 and BT-474R cells with si-ERRα, the cell migration capability was inhibited both cell lines (Figure 2C). As revealed by MTT assays, the suppressive effect of lapatinib on the viability of resistant cancer cells could be considerably attenuated (#*P* < 0.05, ##*P* < 0.01); after ERRα knockdown, the suppressive effects of lapatinib on non-resistant and resistant cells were both significantly enhanced (*P < 0.05, **P < 0.01) (Figure 2D).

As we have mentioned, the overexpression of ERRα within lapatinib-resistant cells activates metabolic adaptations favoring mitochondrial energy metabolism via up-regulated glutamine metabolism, and ROS detoxification that is necessary for cell viability under the treatment stresses [17]. Next, the effects of ERRα knockdown on the GSH/GSSG and ROS production were evaluated in BT-474 and BT-474R cell lines upon lapatinib treatment. We assessed the ratio of reduced glutathione to oxidative glutathione (GSH/GSSG) to monitor cell detoxification. ERRα knockdown resulted in down-regulated GSH level and up-regulated GSSG level, as manifested as the overall reduction of GSH/GSSG ratio upon lapatinib treatment, indicating suppressed cell detoxification and enhanced oxidative damage (Figure 2E,F). Next, the production of ROS was significantly lower in BT-474R cells; after ERRα knockdown, ROS production was significantly increased in both BT-474 and BT-474R cells upon lapatinib treatment (Figure 2G). Then, mitochondrial membrane potential in BT-474 and BT-474R cells was evaluated with JC-1. Mitochondrial membrane potential in BT-474R cells was observably increased when compared with that in BT-474 cells; after ERRα knockdown, mitochondrial membrane potential was notably decreased in both BT-474 and BT-474R cells upon lapatinib treatment (Figure 2H). These data indicate that ERRα knockdown could modulate the metabolic adaptations favoring mitochondrial energy metabolism by up-regulating glutamine metabolism and detoxification capacity of ROS and decreasing mitochondrial membrane potential, so as to make lapatinib-resistant breast cancer cells re-sensitive to lapatinib.

### SHTM2 might be involved in ERRα functions via acting as a target of ERRα

**Figure 3:**
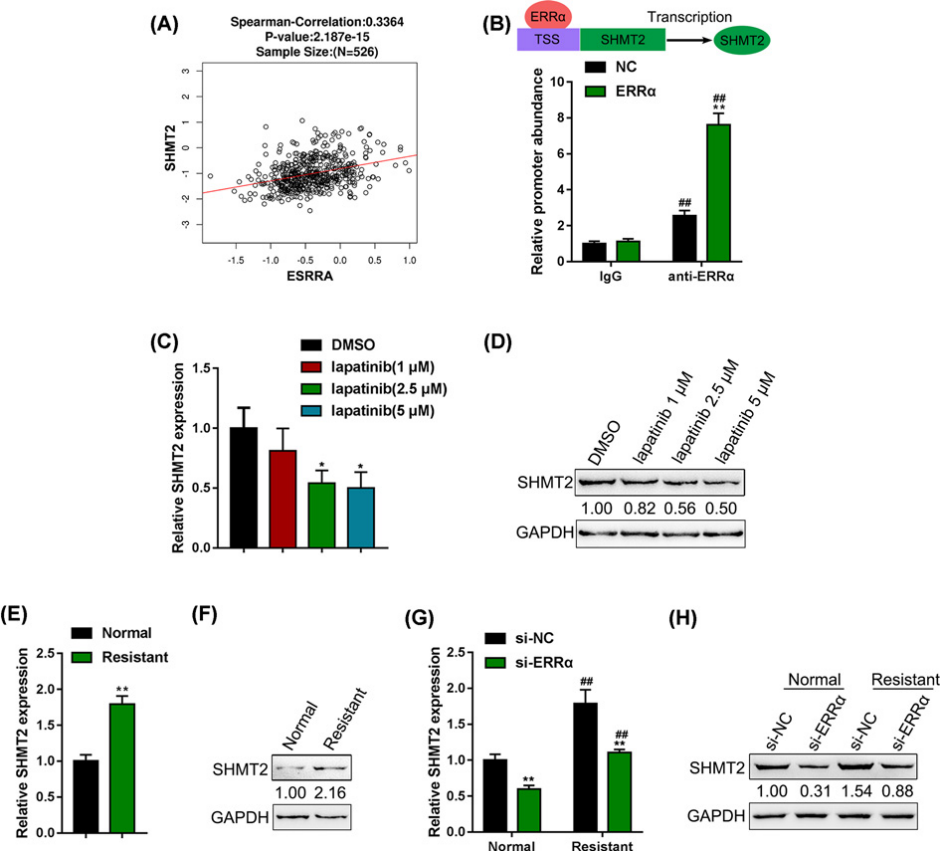
SHTM2 might be involved in ERRα functions via acting as a target of ERRα (**A**) The correlation of ESRRA (ERRα encoding gene) and SHMT2 in tissue samples based on TCGA data. (**B**) A schematic diagram showing the binding of ERRα to SHMT2 promoter region. ChIP assay was performed in NC (negative control) or ERRα-overexpressing vector-transfected BT-474R cells with anti-IgG and anti-ERRα to validate the predicted binding between ERRα and SHMT2. BT-474 cells were treated with lapatinib (0, 1, 2.5 and 5 µM) and examined for (**C**) the expression of SHTM2 by real-time PCR and (**D**) the protein levels of SHTM2 by Immunoblotting. (**E** and** F**) SHMT2 expression and protein levels in BT-474 and BT-474R cells were determined by real-time PCR and immunoblotting. (**G** and **H**) BT-474 and BT-474R cells were transfected with si-ERRα and examined for the expression and protein levels of SHMT2 by real-time PCR and Immunoblotting; *P < 0.05, **P < 0.01, compared with NC group; ##*P* < 0.01, compared with IgG group.

As we have mentioned, ChIP-Atlas/Enrichment Analysis suggested that ERRα could activate the transcription of SHMT2 via targeting its promoter region (data not shown); moreover, the Spearman’s correlation analysis also shows that ESRRA (ERRα encoding gene) expression is positively correlated with SHMT2 expression in tissue samples based on the data from TCGA database (Figure 3A). To determine the predicted binding of ERRα to SHMT2, we conducted ChIP assay in NC (negative control) or ERRα-overexpressing vector-transfected BT-474R cells with anti-IgG and anti-ERRα. As shown in Figure 3B, the level of ERRα binding DNA could be significantly higher compared with that of IgG in anti-ERRα group. These data indicate that ERRα might activate the transcription of SHMT2 via targeting its promoter region.

Next, *in vitro* experiments were performed to investigate the cellular effects of SHMT2 on non-resistant and resistant cancer cells. BT-474 cells were treated with lapatinib (0, 1, 2.5 and 5 µM) and examined for the expression and protein levels of SHTM2. Similar to ERRα, the mRNA and protein expression levels of SHMT2 were significantly down-regulated in 2.5 and 5 μM lapatinib treatment groups when compared with control group (Figure 3C,D). Consistently, SHMT2 expression and protein levels were significantly higher in BT-474R cells, compared with those in BT-474 cells (Figure 3E,F). Next, BT-474 and BT-474R cells were transfected with si-ERRα and examined for the expression and protein levels of SHMT2; in both cell lines, SHMT2 expression and protein levels were significantly suppressed by ERRα knockdown, further confirmed that SHMT2 could be positively regulated by ERRα via binding to its promoter region (Figure 3G,H). These findings indicate that SHTM2 might be involved in ERRα functions in the resistance of cancer cells to lapatinib.

### Dynamic effects of ERRα and SHMT2 on migration capability, cell viability and mitochondrial metabolism in BT-474R cells

**Figure 4:**
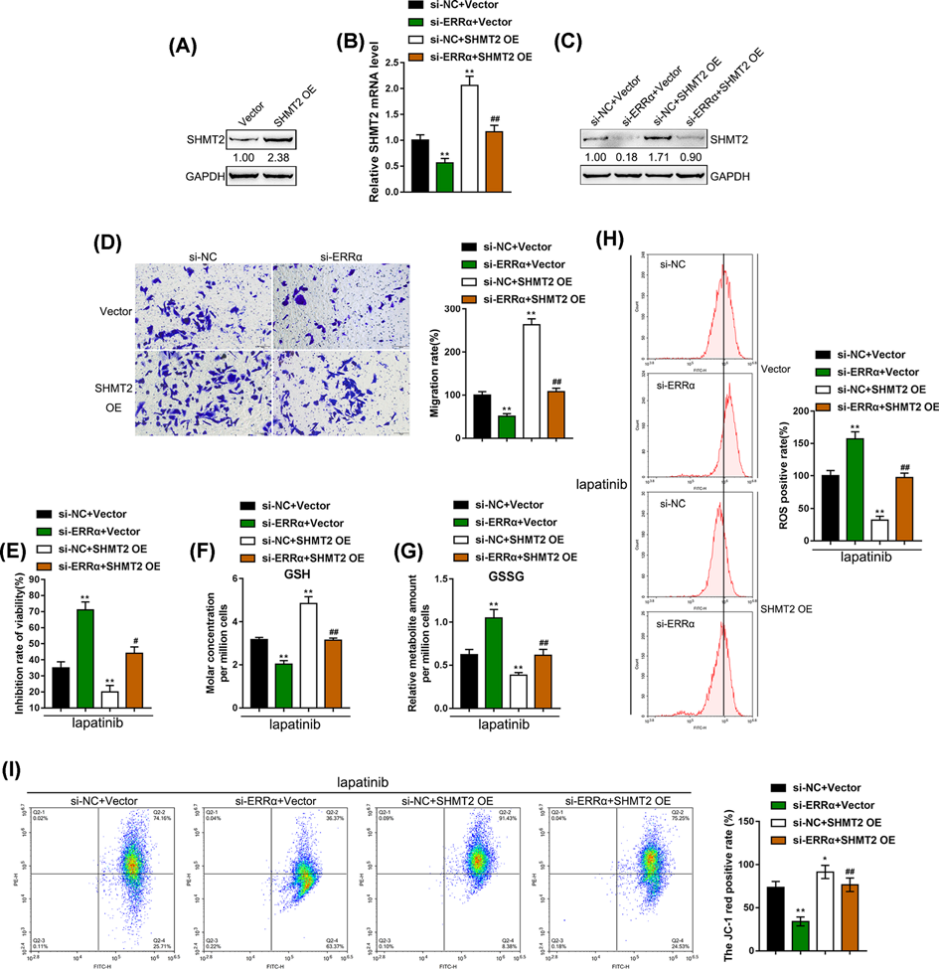
Dynamic effects of ERRα and SHMT2 on migration capability, cell viability and mitochondrial metabolism in BT-474R cells (**A**) SHMT2 overexpression was conducted in BT-474R cells, as confirmed by Immunoblotting. BT-474R cells were co-transfected with si-ERRα and SHMT2 OE vectors and examined for (**B**) the expression of SHMT2 by real-time PCR. (**C**) The protein levels of SHMT2 by Immunoblotting. (**D**) The migration capacity by transwell assay. (**E–H**) Under 5 μM lapatinib treatments, the inhibitory rate of cell viability by MTT assays (E). (**F** and** G**) The GSH and GSSG levels were determined by corresponding kits. (H) The ROS production was measured by flow cytometry. (**I**) The mitochondrial membrane potential was detected by JC-1 staining. *P < 0.05, **P < 0.01, compared with si-NC + vector group; ##*P* < 0.01, compared with si-ERRα + vector group.

After confirming the cellular effects ERRα on lapatinib resistance and the binding between ERRα and SHMT2 promoter region, next, we investigated whether ERRα exerts its effects through SHMT2. We transfected SHMT2 OE vector to conduct SHMT2 overexpression in BT-474R cells, and performed Immunoblotting to verify the transfection efficiency (Figure 4A). Next, we co-transfected BT-474R cell lines with si-ERRα and SHMT2 OE vectors, and then evaluated SHMT2 expression. As shown in Figure 4B,C, SHMT2 expression and protein levels could be significantly decreased by ERRα knockdown whereas increased by SHMT2 overexpression; the effects of ERRα knockdown could be significantly reversed by SHMT2 overexpression. Similarly, BT-474R cell migration capability was inhibited by ERRα knockdown, SHMT2 overexpression could reverse the inhibition (Figure 4D).

Next, the cell viability, the ratio of GSH/GSSG and ROS production were determined under lapatinib treatment. ERRα knockdown enhanced the inhibitory effects of lapatinib on BT-474R cell viability; inversely, SHMT2 overexpression had the opposite effects. SHMT2 overexpression significantly attenuated the effects of ERRα knockdown on BT-474R cells upon lapatinib treatment (Figure 4E). Consistently, ERRα knockdown increased ROS production and GSSG levels whereas reduced GSH levels; SHMT2 overexpression reduced ROS production and GSSG levels whereas increased GSH levels (Figure 4F–H). The JC-1 staining results indicated ERRα knockdown decreased mitochondrial membrane potential, whereas SHMT2 overexpression enhanced mitochondrial membrane potential of BT-474R cells. SHMT2 overexpression significantly attenuated the effects of ERRα knockdown (Figure 4I). In summary, ERRα exerts its effects on the resistance of breast cancer to lapatinib via regulating SHMT2.

## Discussion

In the present study, we revealed that 2.5 and 5 µM lapatinib treatment could significantly decrease the expression and protein levels of ERRα and SHMT2; ERRα and SHMT2 expression and protein levels were significantly up-regulated in breast cancer cells, in particularly in breast cancer cells with resistance to lapatinib. ERRα knockdown restored the inhibitory effects of lapatinib on the BT-474R cell viability and migration; in the meantime, ERRα knockdown rescued the production of ROS whereas decreased the ratio of GSH/GSSG upon lapatinib treatment. Via targeting SHMT2 promoter region, ERRα activated the transcription of SHMT2. The effects of ERRα knockdown on BT-474R cells under lapatinib treatment could be significantly reversed by SHMT2 overexpression.

Mitogenic signaling is considered an important driving factor of breast tumorigenesis, while the resistance to tyrosine kinase inhibitor (TKI), such as lapatinib, is a commonly seen problem within the clinical treatment of HER2-amplified breast tumors. ERRα, a major regulatory factor of cellular energy metabolism, has been previously reported to affect the lapatinib-resistance of breast cancer. The re-expression of ERRα restores the abilities of detoxification and prevents ROS level from increasing in resistant cells, maintaining cell viability in response to treatment disorders caused by oxidative stresses [17]. In the present study, we observed that lapatinib treatment significantly suppressed expression of ERRα within breast cancer cells; moreover, a higher ERRα expression was observed in resistant cancer cells, further confirming the effects of ERRα on breast cancer resistance to lapatinib. Consistently, after conducting ERRα knockdown, the inhibitory effects of lapatinib upon the capacity of resistant breast cancer cells to proliferate and to invade were restored. In the meantime, the ROS production was rescued and the ratio of GSH/GSSG was decreased, further confirming that ERRα knockdown modulates the mitochondrial metabolic adaption to make resistant breast cancer cells re-sensitive to lapatinib.

The prognosis cannot be predicted based on the presence of ERRα, but ERRα target gene expression within breast tumors can produce genomic predictors related to disease prognosis [13]. ERRα up-regulates not only the recruitment to breast cancer biomarker gene TFF1 promoter, but also its expression upon breast cancer cell treatment with epidermal growth factor (EGF) [29]. Likewise, ERBB2 signaling activation within breast cancer cells enhances ERRα target gene expression [30]. Notably, some ERRα target genes do not respond to the stimulation of growth factor, which indicates that even ERRα can be responsive to the inputs of growth factor signaling, the activation mechanisms should be specific to target genes. In the present study, bioinformative and experimental analyses revealed that ERRα activates the transcription and increases the protein level of SHMT2 via targeting its promoter region. More importantly, SHTM2 expression was significantly down-regulated by lapatinib treatment; a higher SHTM2 expression was also observed in lapatinib-resistant breast cancer cells, indicating the effect of SHMT2 on breast cancer resistance to lapatinib.

Reportedly, SHMT2 is considered an important regulator within the serine/glycine metabolism pathway [31,32]; it can be concluded that changes in serine/glycine metabolism characteristics by SHMT2 are related to the maintenance of cancer cell proliferation [33–35]. Lee et al. [36] drew recurrent amplification regions within a large number of primary human cancers and determined that SHMT2 could be essential for the viability of cancer cells. As further evidence, Wang et al. [37] confirmed that SHMT2 overexpression enhanced the growth of gliomas. Lin et al. [38] revealed that SHMT2 expression could be increased within colon cancer tissue samples; in addition, SHMT2 silence could inhibit serine/glycine metabolism to suppress the proliferation of colon cancer cells. In the present study, SHTM2 overexpression exerted opposing effects to those of the ERRα knockdown on the cell proliferation, cell migration, ROS production and the ratio of GSH/GSSG in resistant breast cancer cells upon lapatinib treatment. More importantly, the effects of ERRα knockdown could be significantly reversed by SHTM2 overexpression, indicating that ERRα exerts its effects on breast cancer resistance to lapatinib via activating the transcription of SHTM2.

In conclusion, we demonstrate that ERRα knockdown suppresses the detoxification and the mitochondrial metabolic adaption in breast cancer cells with resistance to lapatinib; ERRα activates SHMT2 transcription via targeting its promoter region, therefore exerting its functions in lapatinib-resistant breast cancer cells. SHMT2 inhibitor has emerged as an effective adjuvant agent for breast cancer treatment using lapatinib, which needs further *in vivo* and clinical investigation.

## Abbreviations

EGF: epidermal growth factor
ERRα: estrogen-related receptor α
GSH: glutathione
ROS: reactive oxygen species
SHMT2: serine hydroxymethyltransferase 2
